# Multi-modal feature selection with anchor graph for Alzheimer's disease

**DOI:** 10.3389/fnins.2022.1036244

**Published:** 2022-11-09

**Authors:** Jiaye Li, Hang Xu, Hao Yu, Zhihao Jiang, Lei Zhu

**Affiliations:** ^1^School of Computer Science and Engineering, Central South University, Changsha, China; ^2^College of Computer Science and Electronic Engineering, Hunan University, Changsha, China; ^3^College of Information and Intelligence, Hunan Agricultural University, Changsha, China

**Keywords:** Alzheimer's disease, anchor graph, local structure, multi-modal, feature selection

## Abstract

In Alzheimer's disease, the researchers found that if the patients were treated at the early stage of the disease, it could effectively delay the development of the disease. At present, multi-modal feature selection is widely used in the early diagnosis of Alzheimer's disease. However, existing multi-modal feature selection algorithms focus on learning the internal information of multiple modalities. They ignore the relationship between modalities, the importance of each modality and the local structure in the multi-modal data. In this paper, we propose a multi-modal feature selection algorithm with anchor graph for Alzheimer's disease. Specifically, we first use the least square loss and *l*_2,1_−norm to obtain the weight of the feature under each modality. Then we embed a modal weight factor into the objective function to obtain the importance of each modality. Finally, we use anchor graph to quickly learn the local structure information in multi-modal data. In addition, we also verify the validity of the proposed algorithm on the published ADNI dataset.

## 1. Introduction

Alzheimer's disease is a degenerative disease of the central nervous system in the elderly. It is one of the most common chronic diseases in human aging. Its clinical manifestations are memory impairment, aphasia, impairment of abstract thinking and computing power, personality, and behavior changes, etc.. At present, it can not be cured, only through comprehensive treatment to delay the development. However, the current study shows that if effective treatment is carried out in the early stage of Alzheimer's disease (i.e., mild cognitive impairment), further deterioration of the disease can be prevented. Therefore, how to accurately judge which stage the patient is in is very important.

On the one hand, in the information data about patients with Alzheimer's disease, due to personal privacy and other reasons, the data volume is relatively small, but the data dimension is relatively high. For example, in references Jie et al. (2013) and Liu et al. (2014), the amount of data is small, but their dimensions are indeed hundreds of thousands. On the other hand, the data on patients with Alzheimer's disease are often multi-modal, i.e., it includes three different modalities: magnetic resonance imaging (MRI), positron emission tomography (PET), and cerebrospinal fluid (CSF) biomarkers. Different modalities have different characteristics and functions. Therefore, we not only need to reduce the dimension of the data, but also need to conduct multi-modal analysis of the data (i.e., the relationship between different modalities).

In addition, the current dimension reduction algorithms have high time complexity, i.e., the graph needs to calculate the similarity relationship between all sample points, which leads to a large amount of calculation. For example, Zhang et al. (2017) proposed a multi-modal feature selection algorithm. It uses the traditional graph laplace method to learn the local structure information in the data, and its calculation amount is large. Therefore, for the diagnosis of Alzheimer's disease, we need to carry out effective multi-modal feature selection with low time complexity.

In view of the above problems, this paper proposes a multi-modal feature selection algorithm for Alzheimer's disease diagnosis. Specifically, we first use the data under three modalities (i.e., MRI, PET, and CSF) to perform linear fitting with the labels, respectively, so as to obtain the weight relationship matrix for each modality. Then, we introduce anchor graph to quickly construct the relationship between samples, which can not only reduce the time complexity of the algorithm, but also learn the graph structure information in the data. Finally, we introduce *l*_2,1_ sparse regularization term to obtain the weight of each feature and perform multi-modal feature selection. In addition, the proposed method also considers the relationship between modalities (i.e., the importance of each modality).

The main contributions of this paper are as follows:

- We propose a multi-modal feature selection framework for Alzheimer's disease, which can select important feature subsets to help the early diagnosis and prediction of Alzheimer's disease.
- The anchor graph is embedded in the proposed algorithm, which can reduce the time complexity of the algorithm.
- We apply a new alternative iterative optimization strategy to optimize proposed multi-modal feature selection algorithm. It can make the proposed objective function monotonically decrease until convergence in each iteration.
- For the proposed algorithm, we have carried out a series of experiments on ADNI datasets to verify the validity of the proposed method.

## 2. Related work

In this section, we will introduce some work on Alzheimer's disease from two aspects. That is, 1. Research on Alzheimer's disease with feature selection Algorithm 2. Research on Alzheimer's disease with multi-modal learning technology.

### 2.1. Feature selection for Alzheimer's disease

Alzheimer's disease is the most common dementia disease in the elderly. In 2016, a survey showed that more than 40 million people worldwide suffer from Alzheimer's disease, and this number is expected to double every 20 years. At the same time, the researchers found that if the patients were treated at the early stage of the disease, it could effectively delay the development of the disease. On the other hand, with the development of machine learning and deep learning, researchers use artificial intelligence algorithms to explore and understand the pathogenesis of Alzheimer's disease, thus providing a fast and effective way to explore the disease.

At present, most researchers use classification (Yu et al., 2022), regression, and clustering techniques to predict Alzheimer's disease data (Zhang et al., 2022b). But this ignores the problems caused by the high-dimensional features and redundant features in the data. Therefore, some researchers use feature selection algorithm to preprocess the data. For example, Mahendran and Vincent (2022) proposed an embedded feature selection method for early detection of Alzheimer's disease. Specifically, it first uses quality control, downstream analysis, and normalization to preprocess the data. Then it uses four feature selection algorithms to reduce the dimension of the data, so as to select the most suitable feature selection algorithm. Finally, it uses the deep learning model to classify and predict the reduced dimension data. Gallego-Jutglà et al. (2015) proposed a hybrid feature selection algorithm for early diagnosis of Alzheimer's disease. It classifies each feature by selecting the value range. Rani Kaka and Prasad (2021) used integrated feature selection and multiple support vector machines to predict Alzheimer's disease. Specifically, it first uses adaptive histogram equalization to improve the contrast. Then, it uses fuzzy c-means clustering algorithm to distinguish proteins, cerebrospinal fluid and gray matter. Finally, it uses the feature selection algorithm based on integration to reduce the dimension of the data, so as to classify them by the support vector machine. Chaves et al. (2012) proposed a feature selection algorithm based on association rules for Alzheimer's disease. On the one hand, this method uses principal components analysis (PCA) and partial least squares to reduce the dimension of data. On the other hand, it uses support vector machine to classify data. Thapa et al. (2020) proposed a data-driven technology based on feature selection for early diagnosis of Alzheimer's disease. They pointed out that the combination of neuropsychological scores and MRI features could be helpful for the early diagnosis of Alzheimer's disease. Liu et al. (2019) proposed a deep feature selection algorithm for Alzheimer's disease. This method combines deep learning, feature selection, causal reasoning, and genetic imaging analysis. Chyzhyk et al. (2014) proposed a wrapped feature selection to analyze MRI. This method uses extreme learning machines to train algorithms, thereby extracting original features from brain MRI. Niyas and Thiyagarajan (2022) used fisher scores and greedy search to select features in Alzheimer's disease data. Specifically, it first preprocesses the data. Then it uses fisher's score to rank all features and select the best feature subset. Finally, it uses greedy search to select the sub optimal minimum feature subset.

### 2.2. Multi-modal learning for Alzheimer's disease

The above feature selection algorithms are all for the single-mode Alzheimer's disease dataset. In addition to single-mode, there are multi-modal. Multi-modal learning means that there are more than one source and form of data, and the process of learning in these forms is called multi-modal learning. Multi-modal learning can be divided into five categories: multi-modal representation learning (Zhang C. et al., 2021), modal transformation, alignment (Zhu et al., 2022), multi-modal fusion, and collaborative learning (Li et al., 2019). In this paper, because we use multi-modal feature selection algorithm, we focus on multi-modal feature selection in multi-modal representation learning.

In the study of early diagnosis of Alzheimer's disease, datasets often include three different modalities: magnetic resonance imaging (MRI), positron emission tomography (PET), and cerebrospinal fluid (CSF). Therefore, it is necessary to select multi-modal features of datasets. For example, Zhang Y. et al. (2021) used neuroimaging embedding and feature selection to do early diagnosis of Alzheimer's disease. Specifically, it first uses the *l*_2,1_−norm and multiple hinge losses to obtain the feature weights for each modality. Then it uses *l*_*p*_−norm to fuse the complementary information of each modality. Finally, the convergence of the proposed method is proved theoretically. Shao et al. (2020) proposed a hypergraph based multi-task feature selection algorithm for Alzheimer's disease. Specifically, it first learns the feature subset for each modality separately. Then it selects the common feature subset of all modalities. Finally, it introduces the regularization term of hypergraph to establish the high-order structural relationship between samples. Jie et al. (2013) proposed a feature selection algorithm based on manifold learning for Alzheimer's disease. Specifically, it first performs single task learning in each modality. Then it uses a set of sparse regularization terms to learn the relationship between modalities. Finally, it introduces a laplace regularization term to maintain the geometric distribution in the data structure, so as to make more accurate feature selection. Bi et al. (2020) studied the multi-modal data of Alzheimer's disease by using evolutionary random forest algorithm. Specifically, it randomly selects samples and features to improve the generalization performance of random forest. In addition, it also uses hierarchical clustering to obtain the best decision tree. Jiao et al. (2022) proposed a multi-modal feature selection algorithm based on feature structure fusion for Alzheimer's disease. Specifically, it first calculates the similarity between features to construct the correlation regularization. Then, it uses manifold learning to obtain the local structure information of the data. Finally, it uses two regularization terms combined with low rank learning technology to obtain the feature subset of multi-modal data. Hao et al. (2020) proposed a multi-modal feature selection for Alzheimer's disease. Specifically, it first uses the random forest strategy to obtain the similarity of each mode. Then it uses a group sparse regularization terms and similarity regularization terms to constrain the objective function, so as to obtain the feature subsets for multiple modalities. Finally, it uses multi-kernel support vector machine to classify the reduced dimension data. Zhu et al. (2014) also proposed a multi-modal feature selection method for Alzheimer's disease. Specifically, it first uses canonical correlation analysis to consider the correlation between features. Then it uses the least square loss and *l*_2,1_−norm to select features in multi-modal. Finally, according to the selected features, it performs multi-task learning.

From the above works, we can see that whether it is single-modal feature selection or multi-modal feature selection for Alzheimer's disease. Their core is to select the features that are most helpful for the early diagnosis of Alzheimer's disease, and then use these features to classify and predict the data.

## 3. Method

### 3.1. Notation

In this paper, we use capital bold letters, lowercase bold letters and ordinary letters to represent matrices, vectors, and scalars, respectively. Given a data matrix **X**. **X**_*v*_ represents the data in the *v*-th modality. The *l*_*f*_− norm of the matrix **X** is expressed as $||\text{X}|{|}_{F}={\left(\sum_{j}||{\text{x}}^{j}|{|}_{2}^{2}\right)}^{1/2}$. The *l*_2,1_− norm of **X** is expressed as $||\text{X}|{|}_{2,1}=\sum_{i}\sqrt{{\text{x}}_{i}^{T}{\text{x}}_{i}+\varepsilon }$. In addition, we use **X**^*T*^, **X**^−1^ and *tr*(**X**) to represent the transposition, inverse and trace of matrix **X**, respectively.

### 3.2. Multi-modal learning

In practical applications, datasets often describe the same sample in many forms. For example, when we describe an animal, we can describe it in text, audio, or video. At this time, text, audio, and video can be considered as three modalities. Similarly, in the actual dataset, there are often some multi-modal datasets, and the traditional data mining algorithms can not be well-applied to these datasets. They can only mechanically learn a single modality. Multi-modal learning refers to using a function to model a specific view, and using redundant views of the same input to jointly optimize all functions, ultimately improving the learning effect. The traditional multi-modal feature selection function is as follows:


(1) $$\begin{array}{l} \underset{W_{v}}{\min}{\left\|W_{v}^{T}X_{v}^{T}-Y\right\|}_{F}^{2}+R\left\|W_{v}\right\| \end{array}$$

where $\text{X}\in {\mathbb{R}}^{n\times d_{v}}$ represents the data in the *v*-th modality. ${\text{W}}_{v}\in {\mathbb{R}}^{d_{v}\times k}$ represents the feature selection matrix in the *v*-th modality. **Y** ∈ ℝ^*k*×*n*^ represents the label of all data. *R*||**W**_*v*_|| represents the regular term of **W**_*v*_, which can be the *l*_1_−norm, *l*_2,1_−norm, and *l*_2,*p*_−norm that can realize feature selection.

### 3.3. Anchor graph construction

In graph learning, we often construct graphs according to the similarity calculation between samples (Zhang and Li, 2021). Specifically, we first regard each sample as a node of the graph, and then use the metric function to calculate the similarity or relationship between the samples (Zhang et al., 2022a). Finally, we use the obtained relations or weights in the previous step to construct the edges between nodes (samples), so as to construct the graph structure in the data, so as to learn the local structure or global structure information in the data. The traditional graph learning method is as follows:


(2) $$\begin{array}{l} \underset{s_{i}^{T}1=1,0\leq s_{ij}\leq 1}{\min}\sum_{i,j}\left({\left\|x_{i}-x_{j}\right\|}_{2}^{2}s_{ij}+\eta s_{ij}^{2}\right) \end{array}$$

In Equation (2), it uses the square of Euclidean distance to calculate the distance between the *i*-th sample **x**_*i*_ and the *j*-th sample **x**_*j*_. **S** is a similarity matrix. After **S** is obtained by solving Equation (2), We can obtain the laplace matrix by **L** = **D**−**S**, so as to learn the local structure information in the data.

Although the above method can learn the graph structure in the data, its time complexity is relatively high, because it needs to calculate the similarity between each sample and all other samples. Therefore, some researchers have proposed anchor point graph construction. Specifically, it first generates anchor points from all the data, and then establishes the similarity matrix between the anchor points and the sample points. If the anchor point is selected by random sampling method, its time complexity is *O*(1). Suppose there are *m* anchor points generated, and the total number of samples is *n*, and each sample has *d* features. The time complexity of generating the similarity matrix is *O*[*nd* log(*m*)]. The formula for constructing anchor point graph is as follows:


(3) $$\begin{array}{l} \underset{z_{i}^{T}1=1,z_{i}\geq 0}{\min}\sum_{j=1}^{m}{\left\|x_{i}-a_{j}\right\|}_{2}^{2}z_{ij}+\eta \sum_{j=1}^{m}z_{ij}^{2} \end{array}$$

where **a**_*j*_ is the generated *j*-th anchor point and **Z** ∈ ℝ^*n*×*m*^ is the similarity matrix. η is an adjustable parameter. According to references Nie et al. (2014) and Nie et al. (2021), we can get the solution of **Z** as:


(4) $$\begin{array}{l} z_{ij}=\frac{d_{i,k+1}-d_{ij}}{kd_{i,k+1}-\sum_{j=1}^{k}d_{ij}} \end{array}$$

where $d_{ij}=\|{\text{x}}_{i}-{\text{a}}_{j}{\|}_{2}^{2}$ and *k* is a non-negative parameter. After obtaining the matrix **Z**, we can obtain the similarity matrix through the following formula:


(5) $$\begin{array}{l} \begin{array}{l} S=Z\Delta^{-1}Z^{T} \end{array} \end{array}$$

where **Δ** is a diagonal matrix whose diagonal elements are $\Delta_{jj}=\overset{n}{\underset{i=1}{\sum }}z_{ij}$. The function of anchor point graph is to reduce the time complexity. After the similarity matrix **S** is obtained by constructing anchor points, it is still necessary to obtain the laplace matrix with **L** = **D**−**S**.

### 3.4. Proposed multi-modal feature selection with anchor graph

Equation (1) enables feature selection unless select the appropriate regular term *R*||**W**_*v*_||. Due to the wide applicability of the *l*_2,1_−norm, in this paper, we choose the *l*_2,1_−norm to limit the weight matrix **W**_*v*_. i.e., the following formula can be further obtained:


(6) $$\begin{array}{l} \underset{W_{v}}{\min}{\left\|W_{v}^{T}X_{v}^{T}-Y\right\|}_{F}^{2}+\alpha {\left\|W_{v}\right\|}_{2,1} \end{array}$$

Although Equation (6) can be used for multi-modal feature selection, it ignores the deviation problem in the process of data fitting. In addition, it does not learn the graph structure information existing in the data. Therefore, we further introduce the deviation term and the graph regularization term, as shown in the following formula:


(7) $$\begin{array}{l} \underset{W_{v},b,\;\theta_{v}}{\min}{\left\|W_{v}^{T}X_{v}^{T}+b1_{n}^{T}-Y\right\|}_{F}^{2}+\alpha {\left\|W_{v}\right\|}_{2,1}\;\\ \;+\beta tr(W_{v}^{T}X_{v}^{T}L_{v}X_{v}W_{v}) \end{array}$$

where **L**_*v*_ is the laplace matrix, **b** is the deviation term, and α and β are adjustable hyperparameters. Since what we are doing is multi-modal feature selection, Equation (7) cannot consider the weight of each modality. Different modalities should have different importance. Therefore, we need learn the weight of each modality in Equation (7) and further obtain the final objective function, as shown below:


(8) $$\begin{array}{l} \underset{W_{v},b,\theta_{v}}{\min}{\left\|W_{v}^{T}\Theta_{v}X_{v}^{T}+b1_{n}^{T}-Y\right\|}_{F}^{2}+\alpha {\left\|W_{v}\right\|}_{2,1}\\ \;+\beta tr(W_{v}^{T}X_{v}^{T}L_{v}X_{v}W_{v})\\ s.t.{\left[\theta_{1};\theta_{2};\cdots ;\theta_{v}\right]}^{T}1_{d}=1,\theta \geq 0 \end{array}$$

To sum up, Equation (8) improves multi-modal feature selection from three aspects: 1. The weight of each modality, i.e., **Θ**_*v*_, is considered. 2. The deviation problem in the process of data fitting is considered. 3. Using anchor graph construction to improve the slow learning speed of traditional graph structure.

### 3.5. Optimization

In this section, we optimize the proposed objective function, i.e., Equation (8), by alternating iterations.

**Update** b **by Fixing** W_***v***_ **and** ***θ***_***v***_.

When **W**_*v*_ and ***θ***_*v*_ are fixed, we can obtain the following formula:


(9) $$\begin{array}{l} \underset{b}{\min}{\left\|W_{v}^{T}\Theta_{v}X_{v}^{T}+b1_{n}^{T}-Y\right\|}_{F}^{2} \end{array}$$

Next, we take the derivative of **b** with Equation (9) and make the derivative zero, as follows:


(10) $$\begin{array}{l} \frac{\partial {\left\|W_{v}^{T}\Theta_{v}X_{v}^{T}+b1_{n}^{T}-Y\right\|}_{F}^{2}}{\partial b}=0 \end{array}$$

Further, Equation (10) is equivalent to the following equation:


(11) $$\begin{array}{l} 2W_{v}^{T}\Theta_{v}X_{v}^{T}1_{n}+2b1_{n}^{T}1_{n}-2Y1_{n}=0 \end{array}$$

Through Equation (11), we can obtain the solution of **b** as follows:


(12) $$\begin{array}{l} b=\frac{1}{n}(Y1_{n}-W_{v}^{T}\Theta_{v}X_{v}^{T}1_{n}) \end{array}$$

**Update** W_***v***_ **by Fixing** b **and** ***θ***_***v***_.

When **b** and ***θ***_*v*_ are fixed, Equation (8) can be converted into the following equation:


(13) $$\begin{array}{l} \underset{W_{v}}{\min}{\left\|W_{v}^{T}\Theta_{v}X_{v}^{T}+b1_{n}^{T}-Y\right\|}_{F}^{2}+\alpha {\left\|W_{v}\right\|}_{2,1}\\ \;+\beta tr(W_{v}^{T}X_{v}^{T}L_{v}X_{v}W_{v}) \end{array}$$

On the one hand, since $||{\text{W}}_{v}|{|}_{2,1}=\overset{d_{v}}{\underset{i=1}{\sum }}{\left||{\text{w}}_{vi}|\right|}_{2}$ and ||**w**_*vi*_||_2_ are likely to be 0, this will cause Equation (13) to be non differentiable. Therefore, we introduce a sufficiently small constant ε to solve this problem, i.e., replace ||**w**_*vi*_||_2_ with $\sqrt{{\text{w}}_{vi}^{T}{\text{w}}_{vi}+\varepsilon }$. On the other hand, **L**_*v*_ = **D**_*v*_ − **S**_*v*_, where **L**_*v*_ is a laplace matrix, **D**_*v*_ is a degree matrix, and **S**_*v*_ is a similarity matrix. Since we use anchor point graph construction, ${\text{S}}_{v}={\left(\text{B}{\text{B}}^{T}\right)}_{v}$, where $\text{B}=\text{Z}{\text{Δ}}^{-\frac{1}{2}}$. For the degree matrix **D**_*v*_, its diagonal element value is:


(14) $$\begin{array}{l} D_{ii}=\sum_{sj}Z_{is}{\left(\Delta_{ss}\right)}^{-1}Z_{js}=\sum_{s}Z_{is}=1 \end{array}$$

Therefore, we can get the degree matrix **D**_*v*_ = **I**_*v*_. Further, Equation (13) may be written as the following equation:


(15) $$\begin{array}{l} \underset{W_{v}}{\min}{\left\|W_{v}^{T}\Theta_{v}X_{v}^{T}+b1_{n}^{T}-Y\right\|}_{F}^{2}\\ \;+\alpha \sum_{i=1}^{d_{v}}\sqrt{w_{vi}^{T}w_{vi}+\varepsilon }+\beta tr(W_{v}^{T}X_{v}^{T}(I_{v}-{\left(BB^{T}\right)}_{v})X_{v}W_{v}) \end{array}$$

Further, we use Equation (15) to find the derivative of **W**_*v*_ and let the derivative be 0 to obtain the following formula:


(16) $$\begin{array}{l} \frac{\partial \left(\begin{array}{l} {\left\|W_{v}^{T}\Theta_{v}X_{v}^{T}+b1_{n}^{T}-Y\right\|}_{F}^{2}+\alpha \sum_{i=1}^{d_{v}}\sqrt{w_{vi}^{T}w_{vi}+\varepsilon }\\ +\beta tr(W_{v}^{T}X_{v}^{T}(I_{v}-{\left(BB^{T}\right)}_{v})X_{v}W_{v}) \end{array}\right)}{\partial W_{v}}=0 \end{array}$$

Equation (16) is equivalent to the following equation:


(17) $$\begin{array}{l} 2\Theta_{v}X_{v}^{T}X_{v}\Theta_{v}^{T}W_{v}+2\Theta_{v}X_{v}^{T}1_{n}b^{T}-2\Theta_{v}X_{v}^{T}Y^{T}\\ +2\alpha N_{v}W_{v}+2\beta X_{v}^{T}(I_{v}-{\left(BB^{T}\right)}_{v})X_{v}W_{v}=0 \end{array}$$

where the value of each element in **N**_*v*_ is:


(18) $$\begin{array}{l} N_{vii}=\frac{1}{2\sqrt{w_{vi}^{T}w_{vi}+\varepsilon }} \end{array}$$

According to Equation (17), we can obtain the closed form solution of **W**_*v*_ as:


(19) $$\begin{array}{l} W_{v}={\left(\begin{array}{l} \Theta_{v}X_{v}^{T}X_{v}\Theta_{v}^{T}+\alpha N_{v}\\ +\beta X_{v}^{T}(I_{v}-{\left(BB^{T}\right)}_{v})X_{v} \end{array}\right)}^{-1}\\ \;(\Theta_{v}X_{v}^{T}Y^{T}-\Theta_{v}X_{v}^{T}1_{n}b^{T}) \end{array}$$

**Update** ***θ***_***v***_ **by Fixing** b **and** W_***v***_.

When **b** and **W**_*v*_ are fixed, we solve ***θ***_*v*_. Since ***θ***_*v*_ is the weight for each modality, we can solve the weight under all modalities at once, i.e., ***θ*** = [***θ***_1_; ***θ***_2_; ⋯ ;***θ***_*v*_]. When **W**_*v*_ is solved, i.e., after **W** = [**W**_1_; **W**_2_; ⋯ ;**W**_*v*_] is obtained. At this time, Equation (8) can be written as follows:


(20) $$\begin{array}{l} \underset{\theta }{\min}{\left\|W^{T}\Theta X^{T}+b1_{n}^{T}-Y\right\|}_{F}^{2}\\ s.t.\theta^{T}1_{d}=1,\theta \geq 0 \end{array}$$

We bring Equation (12) into Equation (20) to further obtain the following equation:


(21) $$\begin{array}{l} \underset{\theta }{\min}{\left\|W^{T}\Theta X^{T}H-YH\right\|}_{F}^{2}\\ s.t.\theta^{T}1_{d}=1,\theta \geq 0 \end{array}$$

where $\text{H}={\text{I}}_{n}-\left(1/n\right)1_{n}1_{n}^{T}$. Through simple transformation, we write Equation (21) in the form of trace, and the following formula can be obtained:


(22) $$\begin{array}{l} \underset{\theta }{\min}\left(tr(\Theta X^{T}HH^{T}X\Theta^{T}WW^{T})-tr(2\Theta X^{T}HH^{T}Y^{T}W^{T})\right)\\ s.t.\theta^{T}1_{d}=1,\theta \geq 0 \end{array}$$

Because **HH**^*T*^ = **H** and **Θ**^*T*^ = **Θ**. Therefore, Equation (22) may be further written as follows:


(23) $$\begin{array}{l} \underset{\theta }{\min}\left(tr(\Theta X^{T}HX\Theta WW^{T})-tr(2\Theta X^{T}HY^{T}W^{T})\right)\\ s.t.\theta^{T}1_{d}=1,\theta \geq 0 \end{array}$$

Next, we introduce the following lemma to solve Equation (23), and lemma 3.5 is as follows:

** Lemma 1**. *If a is diagonal, then tr*(**ABAC**) = **a**^*T*^(**B**^*T*^∘**C**)**a**.

*Proof.*


(24) $$\begin{array}{l} tr(ABAC)=a^{T}diag(BAC)\\ =a^{T}vec\{b_{i}^{T}Ac_{i}\}\\ =a^{T}vec\{{\left(b_{i}\circ c_{i}\right)}^{T}a\}\\ =a^{T}{\left(B^{T}\circ C\right)}^{T}a=a^{T}(B^{T}\circ C)a \end{array}$$

By lemma 3.5, Equation (23) can be written as follows:


(25) $$\begin{array}{l} \underset{\theta }{\min}\theta^{T}\left({\left(X^{T}HX\right)}^{T}\circ (WW^{T})\right)\theta -\theta^{T}diag(2X^{T}HY^{T}W^{T})\\ s.t.\theta^{T}1_{d}=1,\theta \geq 0 \end{array}$$

Further, Equation (25) may be written as the following equation:


(26) $$\begin{array}{l} \underset{\theta }{\min}\;\theta^{T}Q\theta -\theta^{T}s\\ s.t.\theta^{T}1_{d}=1,\theta \geq 0 \end{array}$$

where


(27) $$\begin{array}{l} \{\begin{array}{l} Q=(X^{T}H^{T}X)\circ (WW^{T})\\ \;s=diag(2X^{T}HY^{T}W^{T}) \end{array} \end{array}$$

Next, we use the augmented lagrange multiplier method to solve Equation (26). We first introduce the variable **u** to rewrite Equation (26), as follows:


(28) $$\begin{array}{l} \underset{\theta }{\min}\theta^{T}Q\theta -\theta^{T}s\\ s.t.\theta^{T}1_{d}=1,\theta \geq 0,u=\theta \end{array}$$

Further, we construct the augmented lagrangian function as follows:


(29) $$\begin{array}{l} f(\theta ,u,\mu ,\lambda_{1},\lambda_{2})=\theta^{T}Q\theta -\theta^{T}s+\frac{\mu }{2}{\left\|\theta -u+\frac{1}{\mu }\lambda_{1}\right\|}_{F}^{2}\\ \;+\frac{\mu }{2}{\left(\theta^{T}1_{d}-1+\frac{1}{\mu }\lambda_{2}\right)}^{2}\\ s.t.u\geq 0 \end{array}$$

where μ is a lagrange multiplier. Since the variable **u** is introduced, we still solve Equation (29) by alternating iterative optimization. i.e., when the variable **u** is fixed, Equation (29) is equivalent to the following equation:


(30) $$\begin{array}{l} \underset{\theta }{\min}\frac{1}{2}\theta^{T}E\theta -\theta^{T}g \end{array}$$

where


(31) $$\begin{array}{l} \{\begin{array}{c} E=2Q+\mu I_{d}+\mu 1_{d}1_{d}^{T}\\ g=\mu u+\mu 1_{d}-\lambda_{2}1_{d}-\lambda_{1}+s \end{array} \end{array}$$

Obviously, we can get the solution of **θ** as follows:


(32) $$\begin{array}{l} \theta =E^{-1}g \end{array}$$

When **θ** is fixed, Equation (29) can be written as follows:


(33) $$\begin{array}{l} \underset{u\geq 0}{\min}{\left\|u-(\theta +\frac{1}{\mu }\lambda_{1})\right\|}^{2} \end{array}$$

According to Equation (33), we can obtain the solution of **u** as follows:


(34) $$\begin{array}{l} u=pos(\theta +\frac{1}{\mu }\lambda_{1}) \end{array}$$

The function of *pos*(*x*) is to assign the negative element of *x* to 0. For the reader's understanding, we summarize the pseudo code of the algorithm as shown in Algorithm 1.

**Algorithm 1:**
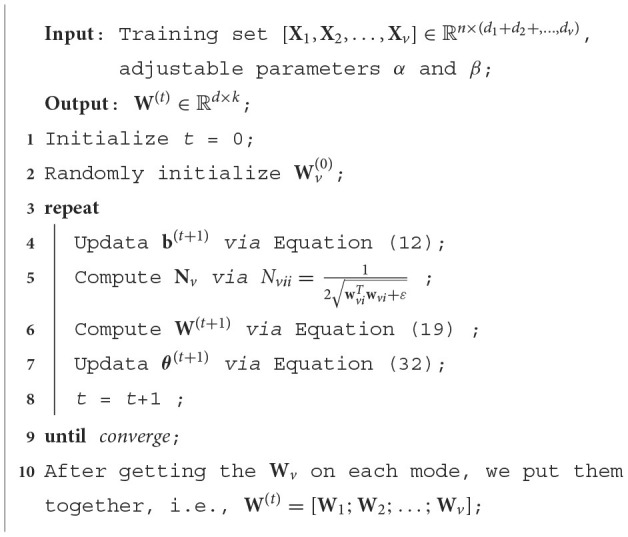
Pseudo code for proposed method.

### 3.6. Convergence analysis

In this section, we prove the convergence of the algorithm. We first introduce the following lemma:

** Lemma 2**. *For any non-zero vector* **x** *and* **y**, *the following formula holds:*


(35) $$\begin{array}{l} {\left\|x\right\|}_{2}-\frac{{\left\|x\right\|}_{2}^{2}}{2{\left\|y\right\|}_{2}}\leq {\left\|y\right\|}_{2}-\frac{{\left\|y\right\|}_{2}^{2}}{2{\left\|y\right\|}_{2}} \end{array}$$

** Theorem 1**. *The value of the proposed objective function monotonically decreases in each iteration until it converges.*

*Proof*. When **b** and ***θ***_*v*_ are fixed, we use ${\text{W}}_{v}^{\left(t\right)}$ and ${\text{W}}_{v}^{\left(t+1\right)}$ to represent the values of **W**_*v*_ at the *t*-th and (*t* + 1)-th iterations, respectively. According to Equation (19), we can obtain the following formula:


(36) $$\begin{array}{l} W_{v}^{\left(t+1\right)}=\underset{W_{v}}{\arg\;\min\;}tr(\left(X_{v}\Theta_{v}^{T}W_{v}^{\left(t\right)}+1_{n}b^{T}-Y^{T}\right)\\ \;\left(W_{v}^{{\left(t\right)}^{T}}\Theta_{v}X_{v}^{T}+b1_{n}^{T}-Y\right))\\ +\alpha tr(W_{v}^{{\left(t\right)}^{T}}N_{v}^{\left(t\right)}W_{v}^{\left(t\right)})+\beta tr(W_{v}^{{\left(t\right)}^{T}}X_{v}^{T}L_{v}X_{v}W_{v}^{\left(t\right)}) \end{array}$$

Further, we can obtain the following formula:


(37) $$\begin{array}{l} tr\left(\left(X_{v}\Theta_{v}^{T}W_{v}^{\left(t+1\right)}+1_{n}b^{T}-Y^{T})(W_{v}^{{\left(t+1\right)}^{T}}\Theta_{v}X_{v}^{T}+b1_{n}^{T}-Y\right)\right)\\ +\alpha tr(W_{v}^{{\left(t+1\right)}^{T}}N_{v}^{\left(t\right)}W_{v}^{\left(t+1\right)})+\beta tr(W_{v}^{{\left(t+1\right)}^{T}}X_{v}^{T}L_{v}X_{v}W_{v}^{\left(t+1\right)})\\ \leq tr\left(\left(X_{v}\Theta_{v}^{T}W_{v}^{\left(t\right)}+1_{n}b^{T}-Y^{T})(W_{v}^{{\left(t\right)}^{T}}\Theta_{v}X_{v}^{T}+b1_{n}^{T}-Y\right)\right)\\ +\alpha tr(W_{v}^{{\left(t+1\right)}^{T}}N_{v}^{\left(t\right)}W_{v}^{\left(t\right)})+\beta tr(W_{v}^{{\left(t\right)}^{T}}X_{v}^{T}L_{v}X_{v}W_{v}^{\left(t\right)}) \end{array}$$

Equation (37) may be rewritten as follows:


(38) $$\begin{array}{l} tr\left(\left(X_{v}\Theta_{v}^{T}W_{v}^{\left(t+1\right)}+1_{n}b^{T}-Y^{T})(W_{v}^{{\left(t+1\right)}^{T}}\Theta_{v}X_{v}^{T}+b1_{n}^{T}-Y\right)\right)\\ +\alpha \sum_{i=1}^{d_{v}}\left({\left\|{\left(W_{v}^{\left(t+1\right)}\right)}_{i}\right\|}_{2}+\frac{{\left\|{\left(W_{v}^{\left(t+1\right)}\right)}_{i}\right\|}_{2}^{2}}{2{\left\|{\left(W_{v}^{\left(t\right)}\right)}_{i}\right\|}_{2}}-{\left\|{\left(W_{v}^{\left(t+1\right)}\right)}_{i}\right\|}_{2}\right)+\beta tr(W_{v}^{{\left(t+1\right)}^{T}}X_{v}^{T}L_{v}X_{v}W_{v}^{\left(t+1\right)})\text{​​​​​​​​​​​​}\\ \leq tr\left(\left(X_{v}\Theta_{v}^{T}W_{v}^{\left(t\right)}+1_{n}b^{T}-Y^{T})(W_{v}^{{\left(t\right)}^{T}}\Theta_{v}X_{v}^{T}+b1_{n}^{T}-Y\right)\right)\\ +\alpha \sum_{i=1}^{d_{v}}\left({\left\|{\left(W_{v}^{\left(t\right)}\right)}_{i}\right\|}_{2}+\frac{{\left\|{\left(W_{v}^{\left(t\right)}\right)}_{i}\right\|}_{2}^{2}}{2{\left\|{\left(W_{v}^{\left(t\right)}\right)}_{i}\right\|}_{2}}-{\left\|{\left(W_{v}^{\left(t\right)}\right)}_{i}\right\|}_{2}\right)+\beta tr(W_{v}^{{\left(t\right)}^{T}}X_{v}^{T}L_{v}X_{v}W_{v}^{\left(t\right)}) \end{array}$$

According to lemma 2, we can get:


(39) $$\begin{array}{l} tr\left(\left(X_{v}\Theta_{v}^{T}W_{v}^{\left(t+1\right)}+1_{n}b^{T}-Y^{T})(W_{v}^{{\left(t+1\right)}^{T}}\Theta_{v}X_{v}^{T}+b1_{n}^{T}-Y\right)\right)\\ +\alpha {\left\|W_{v}^{\left(t+1\right)}\right\|}_{2,1}+\beta tr(W_{v}^{{\left(t+1\right)}^{T}}X_{v}^{T}L_{v}X_{v}W_{v}^{\left(t+1\right)})\\ \leq tr\left(\left(X_{v}\Theta_{v}^{T}W_{v}^{\left(t\right)}+1_{n}b^{T}-Y^{T})(W_{v}^{{\left(t\right)}^{T}}\Theta_{v}X_{v}^{T}+b1_{n}^{T}-Y\right)\right)\\ +\alpha {\left\|W_{v}^{\left(t\right)}\right\|}_{2,1}+\beta tr(W_{v}^{{\left(t\right)}^{T}}X_{v}^{T}L_{v}X_{v}W_{v}^{\left(t\right)}) \end{array}$$

In the (*t* + 1)-iteration, when ${\text{W}}_{v}^{\left(t\right)}$ and $\theta_{v}^{\left(t\right)}$ are fixed, we can obtain the closed form solution of **b**^(*t*+1)^ according to Equation (12). Therefore, it is easy to obtain the following formula:


(40) $$\begin{array}{l} tr\left(\left(X_{v}\Theta_{v}^{{\left(t\right)}^{T}}W_{v}^{\left(t\right)}+1_{n}b^{{\left(t+1\right)}^{T}}-Y^{T})(W_{v}^{{\left(t\right)}^{T}}\Theta_{v}^{\left(t\right)}X_{v}^{T}+b^{\left(t+1\right)}1_{n}^{T}-Y\right)\right)\\ +\alpha {\left\|W_{v}^{\left(t\right)}\right\|}_{2,1}+\beta tr(W_{v}^{{\left(t\right)}^{T}}X_{v}^{T}L_{v}X_{v}W_{v}^{\left(t\right)})\\ \leq tr\left(\left(X_{v}\Theta_{v}^{{\left(t\right)}^{T}}W_{v}^{\left(t\right)}+1_{n}b^{{\left(t\right)}^{T}}-Y^{T})(W_{v}^{{\left(t\right)}^{T}}\Theta_{v}^{\left(t\right)}X_{v}^{T}+b^{\left(t\right)}1_{n}^{T}-Y\right)\right)\\ +\alpha {\left\|W_{v}^{\left(t\right)}\right\|}_{2,1}+\beta tr(W_{v}^{{\left(t\right)}^{T}}X_{v}^{T}L_{v}X_{v}W_{v}^{\left(t\right)}) \end{array}$$

When ${\text{W}}_{v}^{\left(t+1\right)}$ and **b**^(*t*+1)^ are fixed, we can get the following according to Equation (32):


(41) $$\begin{array}{l} tr\left(\left(X_{v}\Theta_{v}^{{\left(t+1\right)}^{T}}W_{v}^{\left(t+1\right)}+1_{n}b^{{\left(t+1\right)}^{T}}-Y^{T})(W_{v}^{{\left(t+1\right)}^{T}}\Theta_{v}^{\left(t+1\right)}X_{v}^{T}+b^{\left(t+1\right)}1_{n}^{T}-Y\right)\right)\\ +\alpha {\left\|W_{v}^{\left(t+1\right)}\right\|}_{2,1}+\beta tr(W_{v}^{{\left(t+1\right)}^{T}}X_{v}^{T}L_{v}X_{v}W_{v}^{\left(t+1\right)})\\ \leq tr\left(\left(X_{v}\Theta_{v}^{{\left(t\right)}^{T}}W_{v}^{\left(t+1\right)}+1_{n}b^{{\left(t+1\right)}^{T}}-Y^{T})(W_{v}^{{\left(t+1\right)}^{T}}\Theta_{v}^{\left(t\right)}X_{v}^{T}+b^{\left(t+1\right)}1_{n}^{T}-Y\right)\right)\\ +\alpha {\left\|W_{v}^{\left(t+1\right)}\right\|}_{2,1}+\beta tr(W_{v}^{{\left(t+1\right)}^{T}}X_{v}^{T}L_{v}X_{v}W_{v}^{\left(t+1\right)}) \end{array}$$

According to Equations (39)–(41), we can finally obtain:


(42) $$\begin{array}{l} tr\left(\left(X_{v}\Theta_{v}^{{\left(t+1\right)}^{T}}W_{v}^{\left(t+1\right)}+1_{n}b^{{\left(t+1\right)}^{T}}-Y^{T})(W_{v}^{{\left(t+1\right)}^{T}}\Theta_{v}^{\left(t+1\right)}X_{v}^{T}+b^{\left(t+1\right)}1_{n}^{T}-Y\right)\right)\\ +\alpha {\left\|W_{v}^{\left(t+1\right)}\right\|}_{2,1}+\beta tr(W_{v}^{{\left(t+1\right)}^{T}}X_{v}^{T}L_{v}X_{v}W_{v}^{\left(t+1\right)})\\ \leq tr\left(\left(X_{v}\Theta_{v}^{{\left(t\right)}^{T}}W_{v}^{\left(t\right)}+1_{n}b^{{\left(t\right)}^{T}}-Y^{T})(W_{v}^{{\left(t\right)}^{T}}\Theta_{v}^{\left(t\right)}X_{v}^{T}+b^{\left(t\right)}1_{n}^{T}-Y\right)\right)\\ +\alpha {\left\|W_{v}^{\left(t\right)}\right\|}_{2,1}+\beta tr(W_{v}^{{\left(t\right)}^{T}}X_{v}^{T}L_{v}X_{v}W_{v}^{\left(t\right)}) \end{array}$$

From Equation (42), we can see that the proposed algorithm is monotonically decreasing and convergent. Thus, theorem 1 is proved.

## 4. Experiment

In this section, we compare the proposed algorithm with six comparison algorithms on three ADNI sub-datasets.

### 4.1. Dataset

Data used in the preparation of this article were obtained from the Alzheimer's Disease Neuroimaging Initiative (ADNI) database^1^. The ADNI was launched in 2003 as a public-private partnership, led by Principal Investigator Michael W. Weiner, MD. The primary goal of ADNI has been to test whether serial magnetic resonance imaging (MRI), positron emission tomography (PET), other biological markers, and clinical and neuropsychological assessment can be combined to measure the progression of mild cognitive impairment (MCI) and early Alzheimer's disease (AD).

We downloaded three sub-datasets from the ADNI website, namely AD vs. NC (sick state vs. normal control), MCI vs. NC (moderate cognitive impairment vs. normal control), and pMCI vs. sMCI (Progress MCI vs. stable MCI). ADNI is the authoritative data center for studying Alzheimer's disease. It was jointly funded by the national institutes of health and the national institute of aging in 2004. It is dedicated to collecting and sorting out the data of Alzheimer's disease patients, tracking the onset process of patients, exploring the changes and causes of the onset process, so as to reveal the pathogenesis of Alzheimer's disease and find a cure. It includes clinical data, magnetic resonance imaging data, positron emission computed tomography data, genetic data, and biological sample data.

In this paper, we obtained basic MRI, PET, and CSF data from 202 experimental subjects (including 51 AD subjects, 52 NC subjects, and 99 MCI subjects). Specifically, we retained 93 features from MRI as the first modality, 93 features from PET as the second modality and three features from CSF as the third modality. Detailed object information is shown in Table 1.

**Table 1:** Demographic information of the subjects.

	**AD**	**NC**	**MCI-C**	**MCI-NC**
	**(51)**	**(52)**	**(43)**	**(56)**
Female/male	18/33	18/34	15/28	17/39
Age	75.2 ± 7.4	75.3 ± 5.2	75.8 ± 6.8	74.8 ± 7.1
Education	14.7 ± 3.6	15.8 ± 3.2	16.1 ± 2.6	15.8 ± 3.2
MMSE	23.8 ± 2.0	29.0 ± 1.2	26.6 ± 1.7	28.4 ± 1.7
adas-Cog	18.3 ± 6.0	12.1 ± 3.8	12.9 ± 3.9	8.03 ± 3.8

The numbers in parentheses denote the number of subjects in each clinical category. (MCI-C, MCI converters; MCI-NC, MCI non-converters; Mean ± SD).

### 4.2. Comparison algorithm

OMVFS (Online unsupervised Multi-View Feature Selection; Shao et al., 2016): this method is a multi-modal feature selection algorithm. It does not store all data, but performs data processing step by step to compress the required data into a matrix. In addition, it also combines graph regularization term, sparse learning and non negative matrix decomposition technology to select features.

K-OFSD (Online Feature Selection based on the Dependency in K nearest neighbors; Zhou et al., 2017): it is a feature selection algorithm for class imbalance data. Specifically, according to the neighborhood rough set theory, it uses the information of k-nearest neighbors to select features, so as to improve the separability between the majority class and the minority class. In addition, it also uses the relationship between labels and features to obtain the importance of each feature.

RLSR (Rescaled Linear Square Regression; Chen et al., 2017): this method is a semi-supervised feature selection algorithm. It scales the regression sparsity of the least squares loss function again by using the scaling factor, so as to obtain the weight of each feature. In addition, it also explains that this method can learn the global structure of data and get sparse solution.

PMFS (Pareto-based feature selection algorithm for multi-label classification; Hashemi et al., 2021): this method is a pareto based feature selection algorithm. Specifically, it first establishes a dual objective optimization model of feature redundancy and feature correlation by using multiple labels. Then, it uses pareto to solve the established model in the previous step. Finally, it verifies the performance of the proposed method in experiments.

MDFS (Embedded feature selection method *via* manifold regularization; Zhang et al., 2019): this method is a multi-label feature selection algorithm. It uses the original features to construct a low dimensional embedding to learn the local structure information of the data. In addition, it embeds *l*_2, 1_−norm into the proposed objective function to select the feature subset.

SDFS (Sparsity Discriminant Feature Selection; Wang et al., 2020): this method is a feature selection algorithm based on *l*_2, 0_−norm. It uses structured sparse subspace constraints to overcome the problem of parameter adjustment. In addition, it also uses the objective function to improve the resolution of the model.

### 4.3. Experimental setup

In this section, we conducted 10-fold cross validation experiments. Specifically, we first carried out comparative experiments on classification accuracy (acc), sensitivity (sen), specificity (spe), and area under curve (auc) of all algorithms on three datasets. Then, we carry out the parameter sensitivity experiment of the proposed algorithm. Finally, we verify the convergence of the proposed algorithm on three datasets. For all algorithms, after obtaining the selected feature subset, we use support vector machine (SVM) to classify them, so as to compare the performance of all algorithms. For parameters α and β. We set their value range as α, β ∈ {10^−3^, 10^−2^, 10, 1, 10, 10^2^, 10^3^}. In addition, we also set the convergence condition of the proposed algorithm as $\frac{\left|obj\left(t+1\right)-obj\left(t\right)\right|}{obj\left(t\right)}\leq 10^{-5}$, where *obj*(*t*) and *obj*(*t*+1) represent the values of the objective function in the *t*-th iteration and the (*t*+1)-th iteration, respectively.

### 4.4. Analysis of experimental results

**Figure 1:**
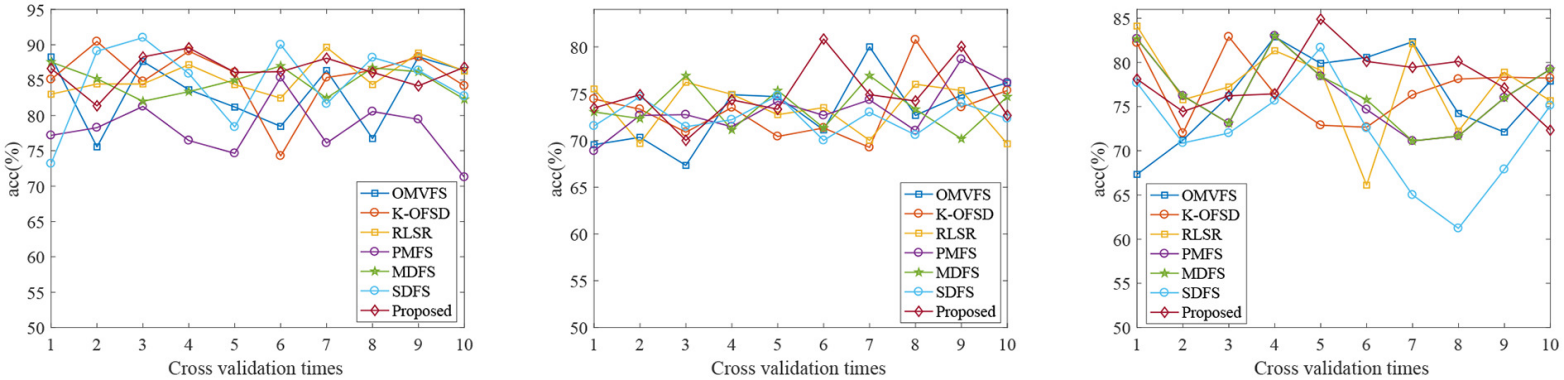
Classification accuracy results of all algorithms on three datasets (From left to right, the results on datasets AD vs. NC, MCI vs. NC, and pMCI vs. sMCI are in turn).

Figure 1 shows the classification accuracy of each fold of all algorithms on three datasets. From Figure 1, we can see that the classification accuracy of all algorithms is not very stable due to the randomness of 10 fold cross validation. From the first and third subgraphs, we can see that PMFS and SDFS have the worst performance. Therefore, we also carried out experiments on the average classification accuracy, average sen, average spe, and average auc of all algorithms on three datasets, as shown in Table 2. From Table 2, we can see that the proposed algorithm achieves the best classification accuracy. Specifically, compared with the worst comparison algorithm PMFS and the best comparison algorithm K-OFSD, the proposed algorithm improves by 8.27 and 0.93%, respectively on the AD vs. NC dataset. On the MCI vs. NC dataset, compared with the poor comparison algorithms OMVFS, K-OFSD, RLSR, and the best comparison algorithm MDFS, the proposed algorithm has improved by 0.71 and 0.34%, respectively. On the pMCI vs. sMCI dataset, compared with the worst comparison algorithm SDFS and the best comparison algorithm MDFS, the proposed algorithm improves by 5.95 and 1.2%, respectively. The reason for this phenomenon is that the proposed algorithm not only considers the relationship between different modes, but also considers the graph structure information in multi-modal data.

**Table 2:** Classification results of all algorithms on the three datasets (%).

**Datasets**	**AD vs. NC**				**MCI vs. NC**				**pMCI vs. sMCI**			
	**acc**	**sen**	**spe**	**auc**	**acc**	**sen**	**spe**	**auc**	**acc**	**sen**	**spe**	**auc**
OMVFS	81.70	81.27	81.70	90.76	73.15	9.08	99.21	38.73	76.32	71.18	82.13	81.76
K-OFSD	85.40	85.12	85.80	92.25	73.15	9.08	99.21	**38.73**	76.42	71.18	82.30	81.71
RLSR	84.24	85.46	83.21	93.08	73.15	9.08	99.21	38.68	76.32	71.18	82.13	81.75
PMFS	78.06	76.95	77.94	86.94	73.28	8.80	99.26	37.24	76.61	**73.18**	80.92	**83.30**
MDFS	84.77	84.99	84.14	92.64	73.52	8.87	99.30	37.84	76.72	**73.18**	81.06	83.13
SDFS	84.65	85.89	85.44	93.49	72.46	**36.08**	87.20	33.21	71.97	67.69	78.99	82.84
Proposed	**86.33**	**87.22**	**86.90**	**93.76**	**73.86**	10.15	**99.52**	37.02	**77.92**	72.34	**82.38**	81.98

The bold values indicate the best experimental results.

**Figure 2:**
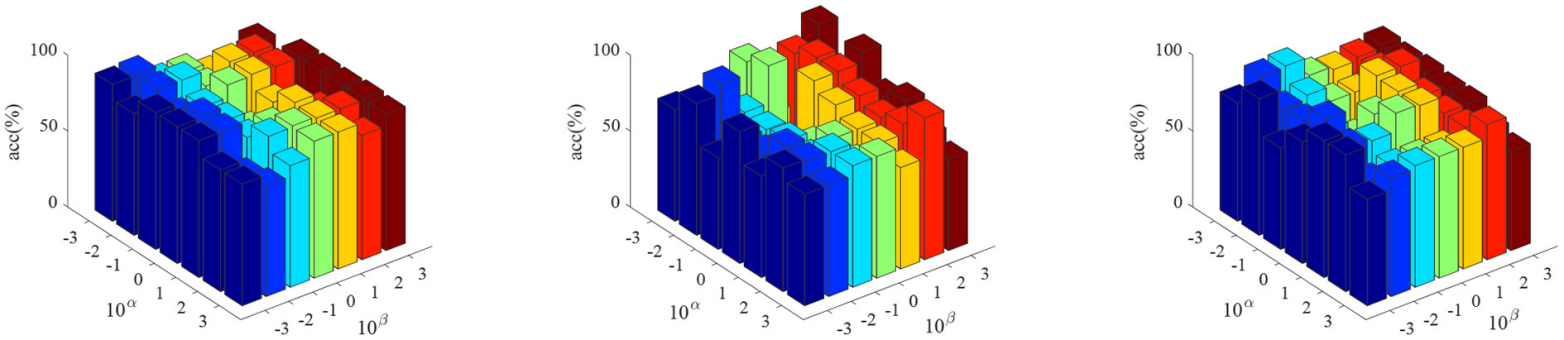
The classification accuracy of the proposed algorithm varies with different parameter values. (From left to right, the results on datasets AD vs. NC, MCI vs. NC, and pMCI vs. sMCI are in turn.)

**Figure 3:**
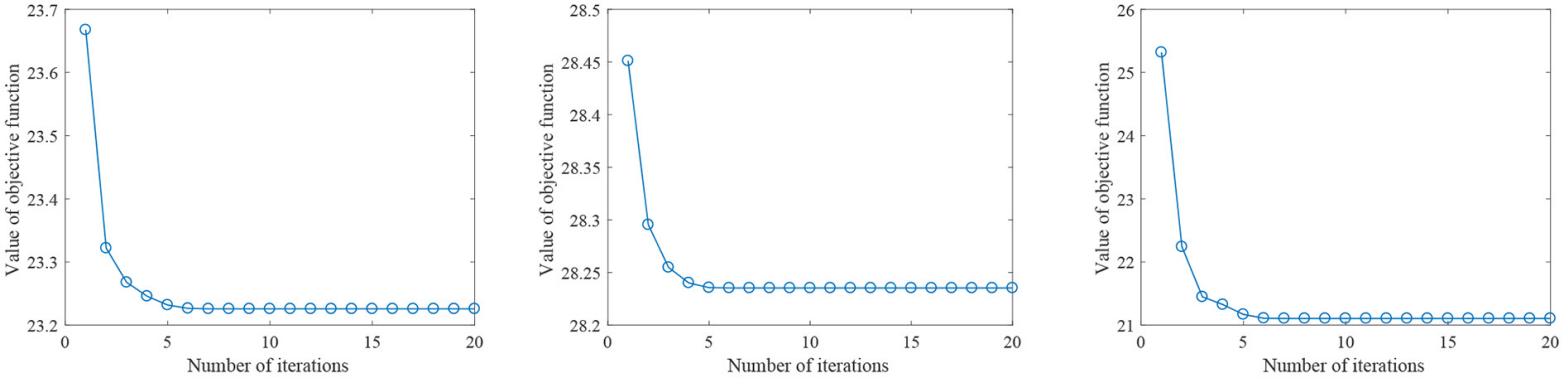
The value of the proposed objective function changes with the number of iterations (From left to right, the results on datasets AD vs. NC, MCI vs. NC, and pMCI vs. sMCI are in turn.)

Figure 2 shows the parameter sensitivity of the proposed algorithm, i.e., the classification accuracy of the proposed algorithm changes with the change of the values of parameters α and β. From Figure 2, we can see that the performance of the proposed algorithm will be affected by the parameter values. Therefore, we need to carefully adjust the values of parameters α and β. In addition, we also conducted the convergence experiment of the algorithm, as shown in Figure 3. From Figure 3, we can see that the proposed algorithm has good convergence. On the three datasets, the convergence was achieved within 10 iterations of the objective function. This shows that the proposed algorithm has fast convergence effect.

## 5. Conclusion

In this paper, we have proposed a multi-modal feature selection algorithm with anchor graph for Alzheimer's disease. It can be used in the early auxiliary diagnosis of Alzheimer's disease. Specifically, we use the least square, *l*_2, 1_−norm and anchor graph regular term to learn the importance of modes, the weight of features and the local structure information of data. In addition, we also prove the convergence of the proposed method. Finally, on the three datasets i.e., AD vs. NC, MCI vs. NC, and pMCI vs. sMCI, we verify the validity of the proposed method and compare it with other advanced comparison algorithms. In the future work, we plan to study new representation methods of multi-modal data, so as to carry out more efficient feature selection for Alzheimer's disease.

## Data availability statement

The original contributions presented in the study are included in the article/supplementary material, further inquiries can be directed to the corresponding author/s.

## Alzheimer's Disease Neuroimaging Initiative

Data used in preparation of this article were obtained from the Alzheimer's Disease Neuroimaging Initiative (ADNI) database (adni.loni.usc.edu). As such, the investigators within the ADNI contributed to the design and implementation of ADNI and/or provided data but did not participate in analysis or writing of this report. A complete listing of ADNI investigators can be found at: http://adni.loni.usc.edu/wp-content/uploads/how_to_apply/ADNI_Acknowledgement_List.pdf.

## Author contributions

JL, HX, HY, and ZJ contributed to conception and design of the study. HY organized the database. JL performed the statistical analysis and wrote the first draft of the manuscript. JL, HX, HY, ZJ, and LZ wrote sections of the manuscript. All authors contributed to manuscript revision, read, and approved the submitted version.

## Funding

This work has been supported in part by the Natural Science Foundation of China under grant 61836016 and National Natural Science Foundation of China under grant 62072166, Natural Science Foundation of Hunan Province under grant 2022JJ40190. Data collection and sharing for this project was funded by the Alzheimer's Disease Neuroimaging Initiative (ADNI) (National Institutes of Health Grant U01 AG024904) and DOD ADNI (Department of Defense award number W81XWH-12-2-0012). ADNI was funded by the National Institute on Aging, the National Institute of Biomedical Imaging and Bioengineering, and through generous contributions from the following: AbbVie, Alzheimer's Association; Alzheimer's Drug Discovery Foundation; Araclon Biotech; BioClinica, Inc.; Biogen; Bristol-Myers Squibb Company; CereSpir, Inc.; Cogstate; Eisai Inc.; Elan Pharmaceuticals, Inc.; Eli Lilly and Company; EuroImmun; F. Hoffmann-La Roche Ltd. and its affiliated company Genentech, Inc.; Fujirebio; GE Healthcare; IXICO Ltd.; Janssen Alzheimer's Immunotherapy Research & Development, LLC.; Johnson & Johnson Pharmaceutical Research & Development LLC.; Lumosity; Lundbeck; Merck & Co., Inc.; Meso Scale Diagnostics, LLC.; NeuroRx Research; Neurotrack Technologies; Novartis Pharmaceuticals Corporation; Pfizer Inc.; Piramal Imaging; Servier; Takeda Pharmaceutical Company; and Transition Therapeutics. The Canadian Institutes of Health Research is providing funds to support ADNI clinical sites in Canada. Private sector contributions are facilitated by the Foundation for the National Institutes of Health (www.fnih.org). The grantee organization is the Northern California Institute for Research and Education, and the study is coordinated by the Alzheimer's Therapeutic Research Institute at the University of Southern California. ADNI data are disseminated by the Laboratory for Neuro Imaging at the University of Southern California.

## Conflict of interest

The authors declare that the research was conducted in the absence of any commercial or financial relationships that could be construed as a potential conflict of interest.

## Publisher's note

All claims expressed in this article are solely those of the authors and do not necessarily represent those of their affiliated organizations, or those of the publisher, the editors and the reviewers. Any product that may be evaluated in this article, or claim that may be made by its manufacturer, is not guaranteed or endorsed by the publisher.

## Footnotes

^1^ adni.loni.usc.edu
